# Non-alcoholic fatty liver disease in diabetes mellitus patients on chronic hemodialysis – A case series addressing cardiovascular and mortality risks

**DOI:** 10.3389/fcdhc.2023.1113666

**Published:** 2023-02-09

**Authors:** Roxana Adriana Stoica, Laura Carina Tribus, Raluca Ioana Marin, Tara David, Carmen Monica Preda, Ioana Cristina Bica, Cristian Serafinceanu

**Affiliations:** ^1^ Department of Diabetes, Nutrition and Metabolic Diseases, University of Medicine and Pharmacy “Carol Davila”, Bucharest, Romania; ^2^ Department of Internal Medicine, University of Medicine and Pharmacy “Carol Davila”, Bucharest, Romania; ^3^ Department of Gastroenterology, Fundeni Clinical Institute, Bucharest, Romania; ^4^ Department of Gastroenterology, University of Medicine and Pharmacy “Carol Davila”, Bucharest, Romania; ^5^ National Institute of Diabetes, Nutrition and Metabolic Diseases, Bucharest, Romania

**Keywords:** NAFLD, diabetes mellitus, hemodialysis, cardiovascular risk, mortality, bioimpedance (BIA), hepatic steatosis index (HSI)

## Abstract

Non-alcoholic fatty liver disease (NAFLD) has an important role in the pathogenesis of cardiovascular diseases in the population with diabetes and it is highly prevalent in end-stage renal disease (ESRD) patients. This case series describes NAFLD associated factors and survival in type 2 diabetes patients (T2DM) who have ESRD treated with hemodialysis. NAFLD prevalence in patients with T2DM and ESRD is 69.2%. A high number of patients (15 out of 18) have obesity evaluated by calculating body mass index (BMI) and bioimpedance measurements. Patients with NAFLD have higher cardiovascular mortality risk, 13 of 18 patients were already diagnosed with coronary heart disease, 6 of 18 had cerebrovascular disease, and 6 of 18 had peripheral artery disease. Fourteen patients were treated with insulin, two patients with sitagliptin (renal adjusted dose of 25mg/day) and two patients with medical nutrition therapy, with an HbA1c ranging from 4.4 to 9.0%. After one-year follow-up 7 of 18 patients died, the causes having roughly equal proportions: myocardial infarction, SARS-CoV2 infection, and pulmonary edema. In conclusion, our population of type 2 diabetic patients with ESRD in hemodialysis had a prevalence of ultrasound-diagnosed NAFLD of 69.2%. Also, this population had a high death rate at one-year follow-up, cardiovascular causes being among the most common.

## Introduction

In the last two decades, the incidence of NAFLD increased significantly, and is a common liver disease spread worldwide (1, 2). It can vary from steatosis to non-alcoholic steatohepatitis and can lead to cirrhosis and finally to hepatocellular carcinoma (2, 3). NAFLD is frequently associated with dyslipidemia, type 2 diabetes, and obesity, and is considered to be a manifestation of metabolic syndrome (4). Primary care systems faced the increase in the burden of the previously mentioned diseases and are responsible for managing cases with low risk of progression of NAFLD, but patients at high risk are referred to gastroenterology (5). When the medical systems are dealing with complex cases, such as the situation of advanced chronic kidney disease (CKD) or dialysis patients, the management of NAFLD includes the nephrologists. The latter consult these categories of patients more often, and recognizing the impact of NAFLD by them is essential for decreasing mortality.

Oxidative stress (6), lipotoxicity, inflammation, and insulin resistance are common pathogenic mechanisms for NAFLD and chronic kidney disease and together are linked to a higher risk of cardiovascular disease (CVD) (2, 7). Also, an alteration in hepatic lipoprotein metabolism occurs, thus accelerating the atherogenic process in patients with a combination of these two pathologies (8).

The decline in glomerular filtration rate (eGFR) evaluated by the annual percent change was higher in patients with NAFLD (−0.79% per year, 95% confidence interval [CI], [−1.31%, −0.27%] versus controls (0.30%, 95% CI [−0.14%, 0.76%]; p = 0.002). A higher fibrosis score, proteinuria, hypertension, smoking status and eGFR below 45 ml/min/1.73 m^2^ are associated with CKD progression in NAFLD (9). A previous systematic review (10) concluded that the influence of NAFLD within the CKD population regarding the major adverse clinical outcomes needs further research. The results of the observational studies included were conflicting, and the effect of NAFLD is difficult to individualize from other metabolic factors (10).

In view of the even smaller number of studies including subjects with end-stage renal disease (ESRD) and NAFLD, more research needs to address this topic. Firstly, NAFLD in patients undergoing hemodialysis (HD) has a high prevalence ranging from 50.5% to 86% depending on the method used for diagnosis (11–14). Secondly, NAFLD increased the chance of CVD by three times in previous studies that included elderly hemodialysis patients (11). Also, the carotid intima-media thickness is a marker of CVD that was significantly higher in subjects with NAFLD and HD (1.430 ± 0.3) compared with CKD alone (1.310 ± 0.2), and normal controls (0.864 ± 0.1) (14). Therefore, this case series aimed to describe the associated NAFLD factors and survival in type 2 diabetes patients (T2DM) who have ESRD.

## Materials and methods

### Design

This is a case series that initially included 26 patients, 11 females (42.3%) and 15 men (57.7%) that have type 2 diabetes mellitus and chronic end-stage renal disease. Patients were evaluated at the National Institute of Diabetes, Nutrition and Metabolic Diseases (INDNBM) N.C Paulescu and the followed-up for12 months (June 2020 - May 2021). This dialysis center is dedicated to patients with diabetes mellitus. The case series was approved by the Ethical Committee of NIDNMD and follows the recommendations of the Helsinki Declaration. All the patients agree to participate by signing the informed consent.

### Clinical and anthropometric parameters

At enrollment, all the patients were evaluated by measuring height, weight, and clinical examination. The demographic and associated disease data were collected from dialysis protocols. Body composition was non-invasively analyzed using the Body Composition Monitor (BCM^®^) device from Fresenius Medical Care. The procedure was performed by applying skin electrodes at the level of the metacarpophalangeal joint, respectively at the level of the metatarsophalangeal joint. Through this procedure parameters such as lean tissue mass-LTM, fat mass-FM, and hydration status (total body water-TBW, overhydration-OH) are determined.

### Biochemical tests

Blood samples were obtained before the HD session: serum creatinine, blood glucose, entry blood urea nitrogen, transaminases (AST- aspartate aminotransferase, ALT- alanine aminotransferase), blood count, lipid profile (total cholesterol/TC, HDL cholesterol/HDLc, LDL cholesterol/LDLc, triglycerides), blood gases.

Using these parameters, we calculated the Hepatic steatosis index (HSI): HSI = 8 × (ALT/AST ratio) + BMI (+2, if female; +2, if DM). This is a tool previously used for screening NAFLD in the general population (cut-off > 36) (15).

### Ultrasound examination of the liver

An ultrasound was performed by the same examiner when presenting for an HD session or if the subject was hospitalized for other reasons (ultrasound equipment Aloka SSD-3500, Aloka Co. LTD). For the diagnosis we used the following ultrasound features: increased hepatorenal echogenicity, vascular blurring of the hepatic or portal vein, and bright hepatic echoes (16).

The inclusion criteria were: diabetes mellitus diagnosis based on the patient’s history and American Diabetes Association guidelines (17)**;** End Stage Renal Disease diagnosis (18); an existing abdominal ultrasound within 6 months with criteria for NAFLD (16).

We excluded subjects with severe cognitive dysfunction, acute and chronic liver pathology (HBs antigen for hepatitis B and VHC antibodies for hepatitis C were tested 3-6 months before).

### Statistics

The patients characteristics were synthesized in a database using SPSS^®^ version 20 (IBM^®^). We considered that a descriptive statistic of the clinical and biological data will be relevant, and given the small number of subjects we expressed the results as a median and interquartile range (IQR). The correlation analysis was performed using the Spearman coefficient. The level of statistical significance was set at p <0.05.

## Results

Of the 26 patients that agree to participate and had complete data, 69.2% had steatosis on US examination. Eighteen subjects with NAFLD, T2DM and ESRD were followed for 12 months in this case series. The female to male ratio was 1:1. The general characteristics of the population are presented in **Table 1**.

**Table 1:** Population general characteristics.

Variable	NAFLD (n=18)
Age (years)	63.00 (14.5)
Duration of diabetes (years)	19.50 (13.25)
Duration of hemodialysis (years)	3.00 (3.25)
Weight (kg)	94.00 (19.5)
BMI (kg/m2)	30.45 (4.62)
FTM (kg)	42.95 (8.75)
LTM (kg)	48.23 (17.7)
HbA1c (%)	7.2 (2.6)
Total cholesterol (mg/dl)	152.91 (65.42)
HDL cholesterol (mg/dl)	33.77 (9.56)
LDL cholesterol calculated (mg/dl)	87.65 (55.43)
Triglycerides (mg/dl)	163.00 (132.6)
AST (U/L)	12.96 (9.29)
ALT (U/L)	15.41 (14.52)
Urinary Albumin Creatinine ratio (mg/g)	1840.85 (1939.7)
Creatinine (mg/dl)	4.81 (1.69)
Blood urea nitrogen (mg/dl)	38.32 (61.76)
Ferritin (ng/ml)	370.10 (222.5)
Serum albumin (g/dl)	3.6 (0.46)

Regarding the complications of diabetes, 14 of 18 patients were diagnosed with retinopathy and 12 of them also had neuropathy. The majority had cardiovascular disease (CVD): coronary heart disease (13 of 18 patients), cerebrovascular disease (6 of 18 patients), and peripheral artery disease (6 of 18 patients).

All HSI values were above 36, with a median of 49.57 (11.74) at first visit, and 50.29 (11.76) at 12 months. There were no significant correlations between HSI and HbA1c, diabetes duration, or dialysis duration.

We observed that patients who receive insulin therapy (14 of 18) have a poorer control with a HbA1c 7.9 (1.8)%, than the others who were treated with oral antidiabetic drugs (sitagliptin 25mg/day) or a controlled diet - HbA1c 5.5 (0.5)%.

At one year follow-up, 7 patients died. The death causes are presented in **Table 2**. The study period overlaps with the COVID-19 pandemic, therefore 2 of 7 patients died after SARS-CoV2 infection.

**Table 2:** Causes of death in NAFLD cases.

Cause	Frequency	Percent
Covid-19	2	11.11
Pulmonary edema	1	5.55
Acute myocardial infarction	2	11.11
Unknown	2	11.11
Total NAFLD cases	18	38.88

**Figure 1:**
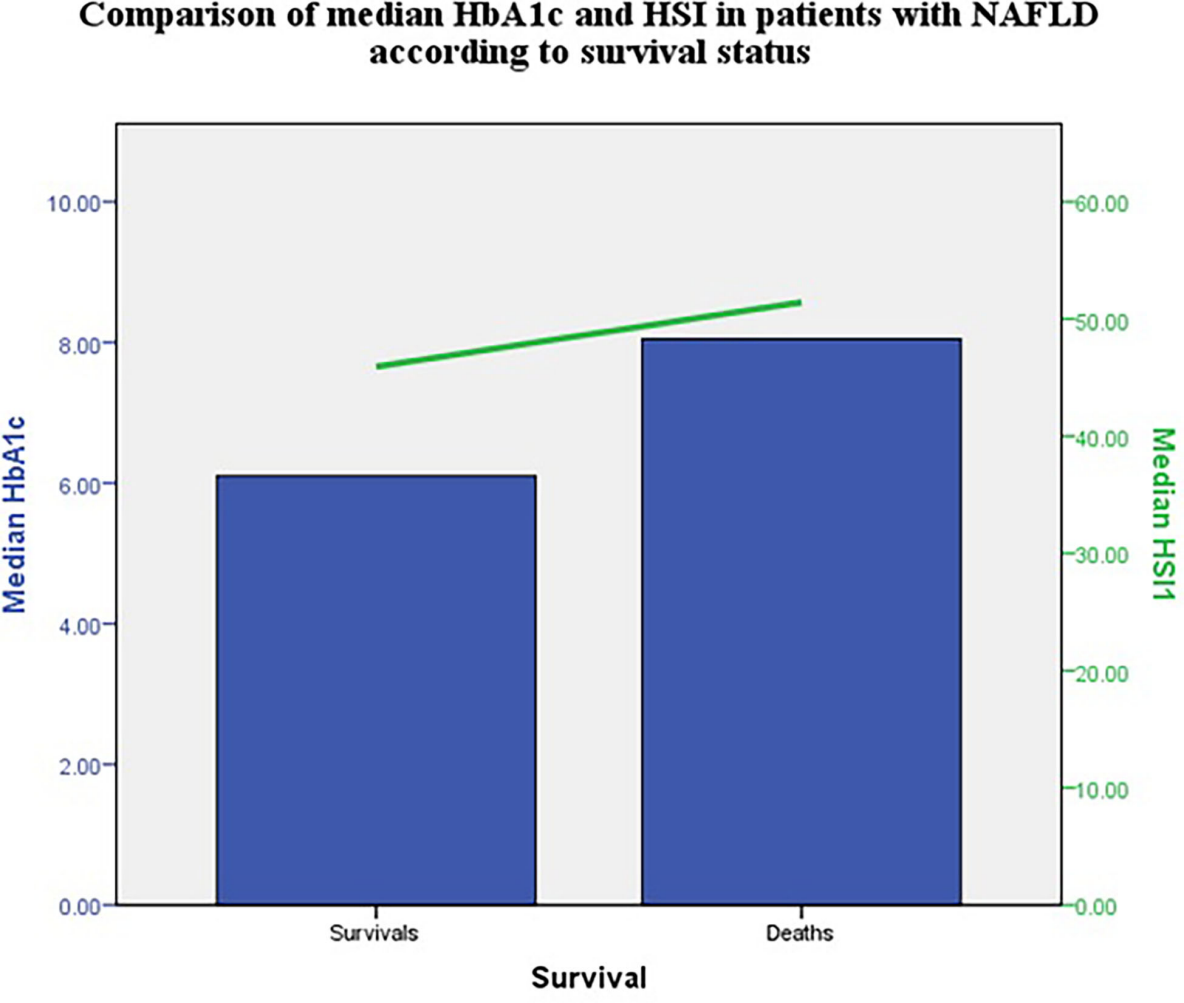
Comparison of median HbA1c and HIS in patients with NAFLD according to survival status.

The median HbA1c and HSI were higher in patients with NAFLD that died compared with those that survived at 12 months (**Figure 1**).

## Discussion

The association of NAFLD with diabetes and ESRD has been observed to increase the occurrence of CVD (6, 11). In our population, 69.2% of subjects were diagnosed with NAFLD and the majority had CVD as a consequence of long-standing diabetes. Although they have an extreme risk of mortality, the target guidelines for lipid profile (17) were not achieved with a median calculated LDLc of 87.65 (55.43) mg/dl, and triglycerides 163.00 (132.6) mg/dL. NAFLD could be the missing link between this atherogenic profile, inflammation, and increased mortality (19).

Median HSI was higher in those that did not survive. This non-invasive index has a good positive predictive value for NAFLD (20) and was associated with carotid atherosclerosis in diabetes (21). Thus, patients with higher values could have higher cardiovascular risk. HSI, together with other steatosis and fibrosis indexes should be evaluated in larger studies.

Inflammation evaluated by high sensitive C reactive protein (hs-CRP) was associated with a poor prognosis in HD patients (22). Also, Capone et al. observed a higher level of high sensitive cTnT in men that was moderately correlated with inflammation (23). Although hs-CRP was not evaluated in our case series, ferritin was available for most of the patients and had higher median levels. Malnutrition-inflammation syndrome could explain an elevated ferritin level above 200ng/ml (24). In previous studies in HD subjects, an U-shape association was observed between ferritin and all-cause mortality (25). Given the increased prevalence of microvascular and macrovascular complications of diabetes, patients have an extremely high mortality risk. Adding NAFLD might be the turning point that hastens the negative prognosis of these patients.

In the 12-month follow-up period 7 deaths were recorded. Regarding the causes of this outcome, SARS-CoV2 infection and myocardial infarction were reported at the same rate. In the HEMO study, cardiac deaths accounted for approximately 40% of mortality on a mean follow-up of 3 years. Patients with cardiac disease at baseline had a relative risk of 2.57 for death (26). In our cases, 13 of 18 had coronary heart disease.

We aimed to determine the prevalence and associated factors with NAFLD in type 2 diabetes patients with ESRD in Romania. The evaluation of body composition, HSI index, and HbA1c dynamics in patients with NAFLD and HD was not described before. Their influence on cardiovascular mortality should be further investigated. The limitations consist of the unavailability of hepatic biopsy, Magnetic Resonance Imaging, or transient elastography with controlled attenuation parameter (12) for confirming steatosis, and the absence of the markers of inflammation.

## Conclusion

The prevalence of NAFLD in our population of type 2 diabetes and ESRD hemodialysis patients was 69.2%. Diabetic patients in hemodialysis with ultrasound-diagnosed hepatic steatosis had a high death rate at one-year follow-up, cardiovascular causes being among the most common. Also, we found that the frail condition of these patients can predispose them to an unfavorable prognosis in the case of SARS-CoV-2 viral outbreaks.

## Data availability statement

The original contributions presented in the study are included in the article/Supplementary Material. Further inquiries can be directed to the corresponding author.

## Ethics statement

The studies involving human participants were reviewed and approved by no 1/19.01.2020 Ethics Committee National Institute of Diabetes Nutrition and Metabolic Diseases. The patients/participants provided their written informed consent to participate in this study.

## Author contributions

All persons who meet authorship criteria are listed as authors, and all authors certify that they have participated sufficiently in the work to take public responsibility for the content, including participation in the concept, design, analysis, writing, or revision of the manuscript. Furthermore, each author certifies that this material or similar material has not been and will not be submitted to or published in any other publication before its appearance in Frontiers in Clinical Diabetes and Healthcare.
